# Relationship Between Sarcopenia, Obesity, Osteoporosis, and Cardiometabolic Health Conditions and Physical Activity Levels in Korean Older Adults

**DOI:** 10.3389/fphys.2021.706259

**Published:** 2021-07-05

**Authors:** Hun-Young Park, Won-Sang Jung, Sung-Woo Kim, Kiwon Lim

**Affiliations:** ^1^Physical Activity and Performance Institute, Konkuk University, Seoul, South Korea; ^2^Department of Sports Medicine and Science, Graduate School, Konkuk University, Seoul, South Korea; ^3^Department of Physical Education, Konkuk University, Seoul, South Korea

**Keywords:** sarcopenia, obesity, osteoporosis, cardiometabolic disease, metabolic syndrome, physical activity level, elderly population

## Abstract

This study aimed to analyze the status of sarcopenia, obesity, osteoporosis, and cardiometabolic disease according to the level of physical activity (PA) among elderly people in Korea. Among the data obtained from the National Health and Nutrition Survey (2008–2011), we analyzed the data of a total of 3,573 Korean elderly people over 65 years of age who were surveyed for dual X-ray absorptiometry (DXA) and PA. Higher levels of PA were associated with a lower prevalence of cardiometabolic disease (χ^2^ = 33.865, *p* < 0.001), osteoporosis (χ^2^ = 94.198, *p* < 0.001), sarcopenia, obesity, and sarcopenic obesity (χ^2^ = 71.828, *p* < 0.001). Above moderate-active PA was associated with lower body weight (*p* < 0.001), body fat mass (*p* < 0.001), and percent body fat (*p* < 0.001), and higher free-fat mass (*p* < 0.001) and appendicular skeletal muscle mass (ASM) (*p* < 0.001) than in low-active PA. In addition, when high-active is the risk factors of cardiometabolic were lower in waist circumference (*p* = 0.001), total cholesterol (TC) (*p* = 0.015), and triglyceride (TG) (*p* < 0.001) than low- and moderate-active PA, and higher in high-density lipoprotein cholesterol (HDL-C) (*p* < 0.001). The prevalence of cardiometabolic diseases was significantly decreased in high-active PA (odds ratio (OR) 0.60, 95% confidence interval (CI) 0.50–0.71); waist circumference (OR 0.85, 95% CI, 0.73–0.99; OR 0.59, 95% CI, 0.50–0.70) and HDL-C (OR 0.76, 95% CI, 0.65–0.88; OR 0.56, 95% CI, 0.47–0.67) significantly improved in moderate- and high-active PA, respectively, and TG (0.67 95% CI, 0.55–0.80) significantly improved in high-active PA. Osteoporosis (OR 0.62, 95% CI, 0.53–0.74; OR 0.46, 95% CI, 0.38–0.55) and sarcopenia (OR 0.77, 95% CI, 0.60–0.98; OR 0.73, 95% CI, 0.57–0.93) were significantly improved in moderate- and high-active PA, respectively. The incidence of obesity (OR 0.47, 95% CI, 0.39–0.57) and sarcopenic obesity (OR 0.47, 95% CI, 0.30–0.75) were significantly decreased in high-active PA. Therefore, we verified a lower prevalence of sarcopenia, osteoporosis, obesity, and cardiac metabolic disease in Korean elderly with more active PA. This suggests that more active PA maybe reduce the prevalence of sarcopenia, osteoporosis, obesity, and cardiometabolic diseases in older adults.

## Introduction

Due to the socio-economic level and the development of medical technology, life expectancy is extended, the elderly are rapidly increasing, and problems related to the elderly are emerging as new social problems (Jung and Park, 2018). Aging causes physical problems, including decreased body function, increased body fat, and reduced muscle mass, as well as a number of social and economic problems, such as lower quality of life and higher rates of chronic diseases (Larsson et al., 2019). Increased body fat and reduced skeletal muscle due to aging are exacerbated by nutritional imbalances and lack of exercise, which has been highlighted in recent studies on sarcopenic obesity, sarcopenia, and obesity (Morley et al., 2014; Batsis and Villareal, 2018). In particular, the elderly who have obesity, sarcopenia, and sarcopenic obesity have a high incidence of cardiometabolic diseases due to decreased daily living ability or exposure to chronic diseases (Jung et al., 2019). Furthermore, their muscular strength, muscular endurance, and balance ability are restricted, which reduces their quality of life (Greco et al., 2019). The decline in muscle strength, joint range of motion (ROM), muscle endurance, and balance ability of the elderly is related to decreases in muscle density, area, and nerve transmission speed with aging, and decreases the ability of the elderly to be active (Larsson et al., 2019). Lack of physical activity (PA) is an independent risk factor associated with increasing chronic diseases and a significantly increased risk of multiple chronic diseases (Strasser, 2013; Jakovljevic, 2018). It is also reported that cardiac output, stroke volume, and heart rate gradually decrease with aging, while the resistance of peripheral blood vessels increases, resulting in continuous heart weakness and blood pressure imbalance, increasing the risk of cardiometabolic dysfunction (Hollmann et al., 2007).

Sarcopenia is the age-related decline in muscle mass, strength, and function; those who develop sarcopenia are at high risk of developing obesity, resulting in sarcopenic obesity (Atkins and Wannamathee, 2020). Older adults have a decrease of approximately 0.5% in the total skeletal muscle mass (SMM) per year and a decrease in muscle strength of approximately 0.3–4.2% (Keller and Engelhardt, 2013). Furthermore, lower extremity strength is reported to decrease by 10–15% every 10 years until age 70 and by 25–40% every 10 years thereafter (Malafarina et al., 2012). Reduced exercise, lack of dietary intake, decreased type 2 muscle fibers in the skeletal muscle, and decreased secretion of insulin-like growth factor-1 (IGF-1) contribute to the development of sarcopenia (Riuzzi et al., 2018; Carter et al., 2019). In addition, sarcopenic obesity leads to an increased risk of developing various diseases, such as hypertension, diabetes, hyperlipidemia, and arteriosclerosis (Atkins and Wannamathee, 2020). Cardiometabolic-related indicators are highly correlated with the risk of major cancers, such as colon cancer, pancreatic cancer, and breast cancer, which are major causes of death in adults (Stefan et al., 2016; Ma et al., 2017; Huh et al., 2018; Hong and Lee, 2020). In particular, the elderly have a correlation with metabolic syndrome (MetS), accompanied by coronary artery diseases, myocardial infarction, and cardiovascular disease. These risk factors increase the proinflammatory state through an increase in C-reactive protein (CRP) and homocysteine levels; and plasminogen activator inhibitor (PAI)-1 and fibrinogen increase blood coagulation and viscosity and interfere with blood flow. In addition, they increase the level of low-density lipoprotein cholesterol (LDL-C), and a high resting heart rate appears due to changes in the sympathetic and parasympathetic nervous systems (Anand et al., 2003; Graziani et al., 2011; Sreckovic et al., 2017; Seo and Hwang, 2020). As described above, obesity and sarcopenia are related to changes in the nervous system (Aagaard et al., 2010), hormonal changes (Morley, 2017), nutritional imbalance (Abiri and Vafa, 2019), decreased PA (Aggio et al., 2016), and continued chronic inflammation (Dalle et al., 2017) and can lead to cardiometabolic-related diseases; thus efforts to maintain a healthy life for the elderly by minimizing changes in body composition due to aging are important.

In order to prevent sarcopenia and obesity and promote health, it is necessary to increase the total energy expenditure (TEE) in regular PA or exercise (Jung et al., 2021). Regular PA reportedly has many health benefits, including a reduction in osteoporosis, sarcopenia, various cancers, mortality, and cardiometabolic diseases, such as obesity, hypertension, type 2 diabetes, and hyperlipidemia (Brown et al., 2012; Fletcher et al., 2018; Greco et al., 2019). PA contributes to a decrease in the overall cardiovascular risk, reducing systolic and diastolic blood pressure and improving left ventricular hypertrophy (Hegde and Solomon, 2015). It can also provide efficient control over MetS and insulin resistance, which are risk factors that increase the sensitivity to severe coronavirus disease-19 (Woods et al., 2020). Many studies have reported that sustained PA and exercise participation may decrease the prevalence of cardiometabolic-related diseases and improve risk factors for adverse health effects (Strasser, 2013; Fletcher et al., 2018; Siedler et al., 2020). PA has many health benefits for older people. However, increasing the PA level in older adults remains challenging. In this study, we aimed to analyze how the PA level in older adults is related to sarcopenia, obesity, osteoporosis, and cardiometabolic health problems, and to understand the optimal level of PA for a positive effect on sarcopenia, obesity, osteoporosis, and cardiometabolic health conditions. Therefore, identifying the level of PA to promote the health of the elderly in Korea was judged to be meaningful by analyzing various relationships between PA levels and sarcopenia, obesity, osteoporosis, and cardiometabolic health conditions, which are problems that appear with aging.

Taken together, the level of PA and exercise participation have a positive impact on the risk factors and prevalence of cardiometabolic diseases. However, the degree of physiological metabolic response and individual adaptation varies depending on the intensity of PA, making standardization of PA and exercise intensity necessary. Therefore, based on the national health nutrition survey data from 2008 to 2011, this study aims to analyze sarcopenia, obesity, osteoporosis, and cardiometabolic-related health conditions according to the level of PA among the elderly in Korea.

## Materials and Methods

### Datasets

This study was analyzed using the 4th and 5th period (2008∼2011) of raw data of the Korea National Health and Nutrition Survey (KNHANES) published by the Korea Disease Control and Prevention Agency (KDCA). For the analysis of sarcopenia, data from 2008 to 2011, when dual X-ray absorptiometry (DXA) tests were conducted, were selected. The KNHANES consists of health interview surveys and health screenings and is conducted in accordance with the Helsinki Declaration. This survey was approved by the Korea Center for Disease Control and Prevention Institutional Review Board (IRB number: 2008-04EXP-01-C, 2009-01CON-03-2C, 2010-02CON-21-C and 2011-02CON-06-C). All participants in the survey signed an informed consent form.

**FIGURE 1:**
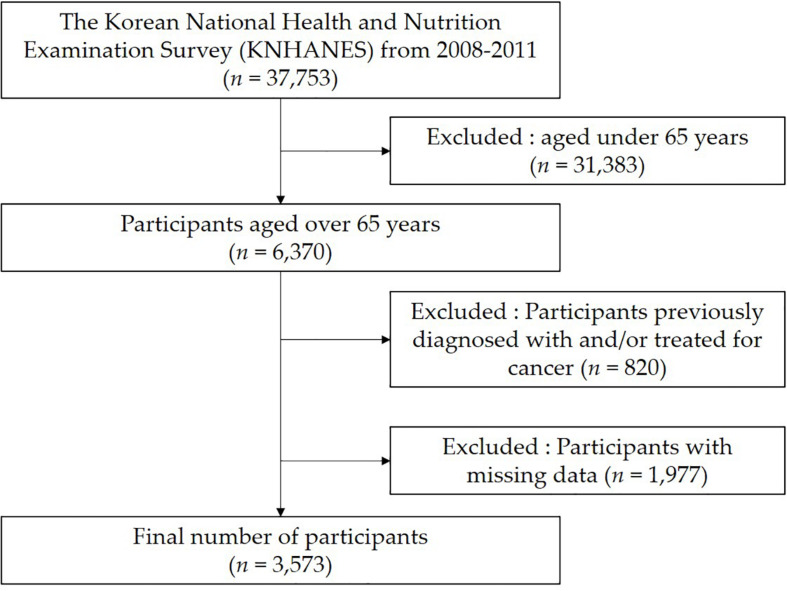
Flow diagram for the selection of study participants.

Between 2008 and 2011, 37,753 health interview surveys and medical examinations were completed. Of these, we excluded 31,383 people under the age of 65 years. Among the remaining 6,370 people, those previously diagnosed with and/or treated for cancer, undergoing surgery for other indications, or with missing data (human measurements, health screening and PA data) were excluded (Figure 1). A total of 3,573 elderly people were finally included in this study.

### Measurement Analysis Data

Data on age, height, weight, fat-free mass, body fat mass, and body fat percentage collected in the National Health and Nutrition Survey were used, and DXA measurements were used for appendicular skeletal muscle mass (ASM), sarcopenia, and osteoporosis. The presence of MetS was determined using measurements of waist circumference, blood pressure, fasting blood glucose levels, triglyceride levels, and HDL-C levels. PA variables were evaluated using the International Physical Activity Questionnaire (IPAQ), and PA was expressed in metabolic equivalent task (MET)-minutes/week. The detailed characteristics of the participants are shown in Table 1.

**TABLE 1:** Baseline characteristics of the subjects.

Variables	Total (*n* = 3,573)	Male (*n* = 1,533)	Female (*n* = 2,040)
Age (years)	71.6 ± 4.6	71.5 ± 4.5	71.8 ± 4.6
Height (cm)	156.9 ± 9.0	165.0 ± 5.7	150.9 ± 5.7
Body weight (kg)	58.6 ± 10.0	63.3 ± 9.4	55.1 ± 9.0
Body mass index (kg/m^2^)	23.8 ± 3.2	23.2 ± 2.9	24.2 ± 3.4
SBP (mmHg)	129.9 ± 17.6	128.0 ± 17.4	131.2 ± 17.6
DBP (mmHg)	75.1 ± 9.8	74.5 ± 9.8	75.5 ± 9.8
Waist circumference (cm)	84.0 ± 9.4	84.9 ± 8.9	83.4 ± 9.7
Fasting glucose (mg/dL)	104.0 ± 26.6	104.9 ± 28.5	103.4 ± 25.0
HDL-C (mg/dL)	45.6 ± 10.9	44.9 ± 11.6	46.1 ± 10.3
TG (mg/dL)	144.6 ± 91.8	141.2 ± 100.4	1,47.2 ± 84.6
Metabolic syndrome (%)	1,583 (44.3)	477 (31.1)	1,106 (54.2)
Education (%)			
≤ Elementary school	2,544 (71.2)	782 (51.0)	1,763 (86.4)
Middle school	414 (11.6)	281 (18.3)	137 (6.7)
High school	407 (11.4)	293 (19.1)	114 (5.6)
≥College	207 (5.8)	179 (11.7)	27 (1.3)
Alcohol (%)	2,448 (68.5)	1,343 (87.6)	1,106 (54.2)
Smoking (%)	1,483 (41.5)	1,268 (82.7)	216 (10.6)
Osteoporosis prevalence (%)	1,341 (37.5)	185 (12.1)	1,179 (57.8)
Sarcopenia prevalence (%)	568 (15.9)	357 (23.3)	210 (10.3)
Obesity prevalence (%)	1,022 (28.6)	164 (10.7)	857 (42.0)
Sarcopenic obesity prevalence (%)	164 (4.6)	63 (4.1)	102 (5.0)

*Values are expressed as means ± standard deviation; SBP, systolic blood pressure; DBP, diastolic blood pressure; HDL-C, high-density lipoprotein cholesterol; TG, triglyceride.*

### Criteria for Sarcopenia, Obesity, and Osteoporosis Diagnosis

For determining the presence of sarcopenia, the ASM, excluding bone and fat, was divided by the square of the height (ASM/height^2^); values < 7.0 kg/m^2^ in males and 5.4 kg/m^2^ in females were considered to indicate sarcopenia (Chen et al., 2020). Obesity was defined by a percent body fat > 28% for males and > 35% for females (Mohammadkhani et al., 2021). Sarcopenic obesity was determined when both sarcopenia and obesity criteria were met (Batsis and Villareal, 2018). Bone condition was classified as normal (T-score ≥ −1.0), osteopenia (−2.5 < T-score < −1.0), and osteoporosis (T-score ≤ −2.5) according to the World Health Organization (WHO) classification standards (Kanis et al., 1994).

### Metabolic Syndrome (MetS)

The diagnosis of MetS was based on the guidelines of the National Cholesterol Education Program-Adult Treatment Panel III and the Korean Society for the Study of Obesity (Seo et al., 2019). If three or more of the following five criteria were met, the participants were classified as having MetS: waist circumference > 90 cm (men) or > 85 cm (women); systolic blood pressure > 130 mmHg or diastolic blood pressure > 85 mmHg; fasting triglyceride (TG) levels > 150 mg/dL; fasting HDL-C levels < 40 mg/dL (men) or < 50 mg/dL (women); and fasting glucose levels > 100 mg/dL.

### Physical Activity Questionnaire

PA was measured using the Korean version of the International Physical Activity Questionnaire short form (IPAQ-SF)- developed by the IPAQ to assess health-related PA. The IPAQ-SF consists of seven questions about the number of days and hours per week spent engaged in vigorous intensity PA, moderate intensity PA, and walking. Based on the IPAQ-SF score conversion method (Craig et al., 2003), the MET (min/week) were calculated as follows:

- Moderate-intensity activity (MET-minutes/week) = 4.0 (MET) × moderate intensity PA minutes × moderate intensity days
- Vigorous-intensity activity (MET-minutes/week) = 8.0 (MET) × vigorous intensity PA minutes × vigorous intensity days
- Walking (MET-minutes/week) = 3.3 (MET) × walking physical activity minutes × walking days
- Total Physical Activity (MET-minutes/week) = vigorous-intensity activity + moderate-intensity activity + walking.

PA levels were classified as low-active (0–599 MET min/week), moderate-active (600–2999 MET min/week), and high-active (> 3,000 MET min/week).

### Statistical Analysis

All analyses were extracted by stratified cluster sampling, and standard plots and weights were utilized for data. To compare the general characteristics, the Chi-square and Student’s *t*-tests were used to analyze categorical and continuous variables, respectively. Continuous variables are presented as means and standard deviations. The normality of distribution of all outcome variables was verified using the Kolmogorov–Smirnov test. One-way analysis of variance was used to compare the differences between the PA levels. The effects of condition on PA levels were analyzed using a mixed procedure. If main effects were statistically significant, *post hoc* Bonferroni correction was performed. Moreover, the relationship between PA levels and MetS was also determined using logistic regression after controlling for covariates using unadjusted and adjusted regression models. Covariate adjustment was implemented in three stages. Model 1, was an unadjusted model; in Model 2, we adjusted for sex, sarcopenia, and obesity; and Model 3 was fully adjusted for sex, sarcopenia, obesity, and osteoporosis. Logistic regression findings are presented as odds ratios (ORs) and their associated 95% confidence intervals (CIs). All statistical analyses were performed using IBM Statistical Package for Social Science (SPSS) version 25.0 for Windows (IBM Corporation, Armonk, NY, United States). Statistical significance was considered at *P* < 0.05.

## Results

### Characteristics of the Study Sample

A total of 3,573 Korean elderly people over 65 years (2,040 women and 1,533 men) was finally included in the study. Their ages ranged from a mean age of 71.6 ± 4.6 years (71.8 ± 4.6 women and 71.5 ± 4.5 men). The prevalence of metabolic syndrome was 44.3% in total, 54.2% for women and 31.1% for men. The education level was the highest in elementary school or below at 71.2%, followed by middle school (11.6%), high school (11.4%), and college or above (5.8%), and men education level was higher than women. Alcohol consumption was 68.5% (women 54.2%, men 87.6%), and smoker was 41.5% (10.6% women, 82.7% men), showing a higher proportion of men than women. The prevalence of osteoporosis was 37.5%, which was higher in women (women 57.8% vs. men 12.1%), and the prevalence of sarcopenia was 15.9%, which was higher in men (women 10.3% vs. men 23.3%). The prevalence of obesity was 28.6% which was higher in women (women 42.0% vs. men 10.7%), and the prevalence of sarcopenic obesity was 4.6%, which was similar (women 5.0% vs. men 4.1%).

### Differences in Physical Characteristics According to the Level of PA

The difference in physical characteristics according to the PA level is men had a high percentage of high-active PA (52.7%), and women had a high percentage of low-active PA (67.5%). The higher the level of PA, the lower the percentage of alcohol consumption and smoking. The prevalence of MetS in the elderly was 44.3% and it lower as the level of PA increased (χ^2^ = 33.865, *p* < 0.001). The number of cardiometabolic-related risk factors lower with increasing PA (χ^2^ = 53.490, *p* < 0.001). The normal elderly and the elderly with osteopenia had an increased percentage of high-active PA, whereas the elderly with osteoporosis had a high percentage of low-active PA (χ^2^ = 94.198, *p* < 0.001). Participants with obesity, sarcopenia, and sarcopenic obesity had a higher percentage of low-active PA (χ^2^ = 71.828, *p* < 0.001; Table 2).

**TABLE 2:** Baseline characteristics by physical activity levels status among Korean elderly.

Characteristics	Categories	MET-min/week			χ^2^	*p*-value
		Low active (*n* = 1,267,%)	Moderate active (*n* = 1,437,%)	High active (*n* = 869,%)		
Gender	Male	412 (32.5)	663 (46.1)	458 (52.7)	96.000	<0.001***
	Female	855 (67.5)	774 (53.9)	411 (47.3)		
Education	≤ Elementary school	970 (77.4)	972 (67.3)	602 (68.9)	45.853	<0.001***
	Middle school	131 (10.5)	171 (11.9)	113 (12.9)		
	High school	105 (8.4)	204 (14.1)	98 (11.2)		
	≥ College	47 (3.8)	98 (6.8)	62 (7.0)		
Alcohol consumption	Yes	451 (35.9)	426 (29.5)	237 (27.2)	21.226	<0.001***
	None	806 (64.1)	1,017 (70.5)	636 (72.8)		
Smoking	Smokers	448 (35.6)	639 (44.3)	404 (46.3)	30.320	<0.001***
	Non-smokers	809 (64.4)	804 (55.7)	469 (53.7)		
Metabolic syndrome	Yes	619 (48.9)	648 (45.1)	315 (36.2)	33.865	<0.001***
	None	648 (51.1)	789 (54.9)	554 (63.8)		
Number of criterion	None of the criteria	82 (6.5)	119 (8.3)	112 (12.9)	53.490	<0.001***
	1 Criteria	247 (19.5)	298 (20.7)	205 (23.6)		
	2 Criteria	319 (25.2)	372 (25.9)	237 (27.3)		
	3 Criteria	309 (24.4)	336 (23.4)	182 (20.9)		
	4 Criteria	233 (18.4)	235 (16.4)	100 (11.5)		
	5 Criteria	77 (6.1)	77 (5.4)	33 (3.8)		
Osteoporosis	Normal	132 (10.7)	268 (18.5)	209 (23.6)	94.198	<0.001***
	Osteopenia	529 (42.7)	671 (46.2)	423 (47.9)		
	Osteoporosis	578 (46.6)	512 (35.3)	251 (28.5)		
Sarcopenia	Normal	640 (50.5)	761 (53.0)	584 (67.2)	71.828	<0.001***
	Sarcopenia	145 (11.4)	167 (11.6)	90 (10.4)		
	Obesity	410 (32.4)	440 (30.6)	171 (19.7)		
	Sarcopenic obesity	72 (5.7)	69 (4.8)	24 (2.8)		

MET, metabolic equivalents. ***p < 0.001.

### Differences in Body Composition and Risk Factors for Cardiometabolic Diseases According to the Level of PA

When examining the difference in body composition according to the level of PA, statistically significant differences were found in all variables, and those with low-active PA showed lower body weight than those with moderate- or high-active PA. Higher levels of PA were associated with a higher free-fat mass (*p* < 0.001) and ASM (*p* < 0.001), and with a lower body fat mass (*p* < 0.001) and percentage body fat (*p* < 0.001). Waist circumference (*p* = 0.001) and TG (*p* < 0.001) were significantly lower, and HDL-C (*p* < 0.001) was higher, when PA levels were high-active PA compared with those in the low- or moderate-active PA conditions. TC (*p* = 0.015) was significantly lower in those with high-active PA compared with that in those with low-active PA. There was no significant difference in SBP, DBP, or fasting glucose (Table 3).

**TABLE 3:** Cardiometabolic risk factors according to physical activity among Korean elderly.

Variables	MET-min/week			*F*-value	*p*-value
	Low active (*n* = 1,267)	Moderate active (*n* = 1,437)	High active (*n* = 869)		
Body weight (kg)	57.4 ± 9.7^a^	59.6 ± 10.2^b^	58.8 ± 10.0^b^	15.865	< 0.001***
Free-fat mass (kg)	39.3 ± 7.3^a^	41.7 ± 8.3^b^	42.7 ± 8.2^*c*^	54.400	< 0.001***
Body fat mass (kg)	17.5 ± 5.8^a^	17.4 ± 5.7^b^	15.7 ± 5.6^*c*^	32.517	< 0.001***
Percent body fat (%)	30.6 ± 7.8^a^	29.3 ± 7.9^b^	26.7 ± 8.0^*c*^	63.993	< 0.001***
ASM (kg)	16.1 ± 3.6^a^	17.3 ± 4.1^b^	18.0 ± 4.1^*c*^	65.730	< 0.001***
ASM/m^2^	6.6 ± 0.9^a^	6.9 ± 1.0^b^	7.1 ± 1.0^*c*^	60.600	< 0.001***
Waist circumference (cm)	84.1 ± 9.5^a^	84.5 ± 9.4^a^	83.0 ± 9.2^b^	6.757	0.001**
SBP (mmHg)	130.6 ± 17.8	129.4 ± 17.4	129.6 ± 17.5	1.757	0.173
DBP (mmHg)	74.8 ± 9.7	75.2 ± 9.8	75.2 ± 9.9	0.738	0.478
Fasting glucose (mg/dL)	104.8 ± 28.8	104.3 ± 25.9	102.6 ± 24.2	1.724	0.178
TC (mg/dL)	193.5 ± 38.2^a^	191.8 ± 36.9^ab^	188.8 ± 35.9^b^	4.238	0.015*
TG (mg/dL)	151.0 ± 95.8^a^	146.4 ± 93.0^a^	132.4 ± 82.1^b^	11.046	< 0.001***
LDL-C (mg/dL)	118.3 ± 35.3	117.4 ± 34.4	115.0 ± 34.2	2.378	0.093
HDL-C (mg/dL)	45.0 ± 10.6^a^	45.1 ± 10.9^a^	47.3 ± 11.2^b^	13.284	< 0.001***

Values are expressed as means ± standard deviation. ASM, appendicular skeletal muscle mass; SBP, systolic blood pressure; DBP, diastolic blood pressure; TC, total cholesterol; TG, triglyceride; LDL-C, low-density lipoprotein cholesterol; HDL-C, high-density lipoprotein cholesterol; AST, aspartate aminotransferase; ALT, alanine aminotransferse; MET, metabolic equivalents. *p < 0.05; **p < 0.01; ***p < 0.001. ^a,b,c^ Different alphabets mean to indicate significant differences between levels of physical activity.

### Odds Ratio of Risk Factors for Osteoporosis, Sarcopenia, Obesity, Sarcopenic Obesity, and Cardiometabolic Risk Factors According to the PA Level (95% CI)

The odds ratio (ORs) of osteoporosis, sarcopenia, obesity, sarcopenic obesity, and cardiometabolic risk factors according to PA levels is as follows. The incidence of MetS was significantly lower in those with high-active PA (0.60, 95% CI, 0.50–0.71); waist circumference (0.85, 95% CI, 0.73–0.99; 0.59, 95% CI, 0.50–0.70) and HDL-C (0.50–0.70; 0.76, 95% CI, 0.65–0.88; 0.56, 95% CI, 0.47–0.67) were significantly improved with moderate- and high-active PA, and TGs (0.67, 95% CI, 0.55–0.80) were significantly improved with high-active PA. The incidence of osteoporosis (0.62, 95% CI, 0.53–0.74; 0.46, 95% CI, 0.38–0.55) and sarcopenia (0.77, 95% CI, 0.60–0.98; 0.73, 95% CI, 0.57–0.93) was significantly lower with moderate- and high-active PA. the incidence of obesity (0.47, 95% CI, 0.39–0.57) and sarcopenic obesity (0.47, 95% CI, 0.30–0.75) was significantly lower with high-active PA (Table 4).

**TABLE 4:** Odds ratio (95% CI) for sarcopenia and cardiometabolic risk factors according to physical activity levels.

Variables	Group	Model 1		Model 2		Model 3	
		OR (95% CI)	*p*-value	OR (95% CI)	*p*-value	OR (95% CI)	*p*-value
MetS	Low active	1.00 (reference)		1.00 (reference)		1.00 (reference)	
	Moderate active	0.86 (0.74–1.00)	0.051	0.93 (0.79–1.10)	0.406	0.89 (0.75–1.06)	0.204
	High active	0.60 (0.50–0.71)	<0.001	0.73 (0.60–0.88)	0.001	0.69 (0.57–0.85)	<0.001
High WC	Low active	1.00 (reference)		1.00 (reference)		1.00 (reference)	
	Moderate active	0.85 (0.73–0.99)	0.039	0.84 (0.70–1.00)	0.061	0.81 (0.66–0.98)	0.032
	High active	0.59 (0.50–0.70)	<0.001	0.68 (0.56–0.84)	<0.001	0.66 (0.53–0.82)	<0.001
High BP	Low active	1.00 (reference)		1.00 (reference)		1.00 (reference)	
	Moderate active	0.99 (0.86–1.16)	0.990	1.00 (0.85–1.20)	0.930	1.02 (0.85–1.22)	0.818
	High active	0.94 (0.79–1.12)	0.514	0.97 (0.81–1.16)	0.721	0.97 (0.81–1.17)	0.758
High Glucose	Low active	1.00 (reference)		1.00 (reference)		1.00 (reference)	
	Moderate active	1.00 (0.86–1.16)	0.983	0.96 (0.82–1.22)	0.609	0.98 (0.83–1.16)	0.808
	High active	0.88 (0.74–1.05)	0.167	0.89 (0.74–1.06)	0.186	0.87 (0.72–1.05)	0.153
High TG	Low active	1.00 (reference)		1.00 (reference)		1.00 (reference)	
	Moderate active	0.95 (0.81–1.11)	0.492	0.96 (0.82–1.12)	0.603	0.94 (0.79–1.11)	0.474
	High active	0.67 (0.55–0.80)	<0.001	0.70 (0.58–0.85)	<0.001	0.70 (0.58–0.86)	<0.001
Low HDL-C	Low active	1.00 (reference)		1.00 (reference)		1.00 (reference)	
	Moderate active	0.76 (0.65–0.88)	<0.001	0.87 (0.74–1.02)	0.084	0.85 (0.72–1.01)	0.063
	High active	0.56 (0.47–0.67)	<0.001	0.68 (0.57–0.82)	<0.001	0.70 (0.57–0.85)	<0.001
Osteoporosis	Low active	1.00 (reference)		1.00 (reference)		1.00 (reference)	
	Moderate active	0.62 (0.53–0.74)	<0.001	0.80 (0.66–0.97)	0.021	0.80 (0.66–0.96)	0.020
	High active	0.46 (0.38–0.55)	<0.001	0.61 (0.48–0.76)	<0.001	0.61 (0.49–0.76)	<0.001
Sarcopenia	Low active	1.00 (reference)		1.00 (reference)		1.00 (reference)	
	Moderate active	0.77 (0.60–0.98)	0.032	0.72 (0.57–0.92)	0.010	0.76 (0.59–0.99)	0.039
	High active	0.73 (0.57–0.93)	0.012	0.59 (0.46–0.76)	< 0.001	0.64 (0.49–0.84)	0.001
Obesity	Low active	1.00 (reference)		1.00 (reference)		1.00 (reference)	
	Moderate active	0.89 (0.76–1.05)	0.158	1.10 (0.93–1.30)	0.267	1.14 (0.95–1.36)	0.160
	High active	0.47 (0.39–0.57)	<0.001	0.60 (0.49–0.74)	<0.001	0.57 (0.46–0.72)	<0.001
SO	Low active	1.00 (reference)		1.00 (reference)		1.00 (reference)	
	Moderate active	0.84 (0.60–1.18)	0.304	0.85 (0.61–1.20)	0.360	0.85 (0.59–1.22)	0.366
	High active	0.47 (0.30–0.75)	0.002	0.48 (0.30–0.78)	0.003	0.49 (0.30–0.81)	0.005

*Values are expressed as odds ratio (95% confidence interval). OR, odds ratio; CI, confidence interval; Mets, metabolic syndrome; WC, waist circumference; BP, blood pressure; TG, triglyceride; HDL, high-density lipoprotein, SO, sarcopenic obesity; Model 1, not adjusted; Model 2, adjusted for sex, sarcopenia, and obesity; Model 3, adjusted for sex, sarcopenia, and obesity, and osteoporosis.*

## Discussion

This study aimed to analyze differences in indicators related to sarcopenia, obesity, osteoporosis, and cardiometabolic risk factors according to the PA level of the elderly in Korea and to confirm their association with the PA level. In this study, as the level of PA increased, the prevalence of cardiometabolic disease, osteoporosis, sarcopenia, obesity, and sarcopenic obesity decreased. Above moderate-active PA levels were associated with lower body weight, body fat mass, and percent body fat and higher free-fat mass and ASM and higher HDL-C than low-active PA. In addition, above high-active PA levels were associated with lower cardiometabolic risk factors, waist circumference, TC, and TG than low- and moderate-active PA. In terms of the odds ratios of osteoporosis, sarcopenia, obesity, sarcopenic obesity, and cardiac metabolic risk factors according to PA levels, the prevalence of cardiometabolic diseases was significantly lower in high-active PA (vs. low- and moderate-active); waist circumference, and HDL-C significantly improved in moderate- and high-active PA (vs. low-active), respectively, and triglycerides significantly improved in high-active PA (vs. low- and moderate-active). Osteoporosis and sarcopenia were significantly improved in moderate- and high-active PA (vs. low-active), respectively. The incidence of obesity and sarcopenic obesity was significantly lower in participants reporting high-active PA (vs. low- and moderate-active).

In this study, looking at the physical characteristics of PA levels, men had a higher rate of high-active PA than low-active PA, while women had a higher rate of low-active PA than high-active PA. Similarly, Grimmer et al. (2019) reported that men showed significantly more PA than women. Amagasa et al. (2017) found that the average intensity of PA (METs) according to gender was similar in the elderly in Japan, but men who weighed more expended more energy through PA than women did. As such, the dissemination of programs to increase PA among elderly women is urgently required. A high percentage of the elderly with MetS reported low-active PA, whereas those without MetS reported a high percentage of high-active PA. In addition, as the level of PA higher, the prevalence of cardiometabolic diseases, osteoporosis, sarcopenia, obesity, and sarcopenic obesity lower. An emerging body of evidence shows that PA plays a preventive role against many diseases such as cardiometabolic diseases, osteoporosis, sarcopenia, obesity, and sarcopenic obesity. Data from our systematic review and meta-analysis, similar to those of previous studies, also show that PA protects against cardiometabolic diseases, osteoporosis, sarcopenia, obesity, and sarcopenic obesity (Moreira et al., 2014; Steffl et al., 2017; Jakovljevic, 2018; Genest et al., 2021; Min et al., 2021).

Examining the factors related to cardiometabolic diseases according to the level of PA, in this study, high-active PA had a positive effect on waist circumference, TG, and HDL-C. Participation in moderate and high-intensity PA is known to have a positive effect on the risk factors and incidence of cardiometabolic diseases (Brown et al., 2012; Strasser, 2013; Fletcher et al., 2018). According to a previous study, participants engaged in high-intensity PA (≥ 60 min per week) had a 63% lower incidence of MetS than those not engaged in high-intensity PA (Laaksonen et al., 2002). In addition, a study of Koreans also reported a significantly lower incidence of MetS in groups participating in regular PA (Jung et al., 2020). Recently, Lacroix et al. (2019) reported that middle-aged and elderly women can significantly reduce the risk of cardiovascular disease by performing low-intensity PA, such as gardening, walking in the park, and folding laundry. As in previous studies, a greater benefit of PA was obtained by increasing PA of moderate or vigorous intensity. However, Serrano-Sánchez et al. (2019) reported that even in those with a prolonged sedentary lifestyle, an increase in light PA can contribute to improving MetS, with beneficial changes in the lipid profile, blood pressure, and insulin resistance. Thus, suggesting ways to increase PA in the elderly is considered important because it can lead to a decrease in cardiometabolic factors.

Finally, in terms of the ORs of osteoporosis, sarcopenia, obesity, sarcopenic obesity, and cardiometabolic risk factors according to PA levels, the prevalence of cardiometabolic diseases was significantly lower in high-active PA (vs. low- and moderate-active); waist circumference, and HDL-C significantly improved in moderate- and high-active PA (vs. low-active), respectively, and triglycerides significantly improved in high-active PA (vs. low- and moderate-active). Osteoporosis and sarcopenia were significantly improved in moderate- and high-active PA (vs. low-active), respectively. Obesity and sarcopenic obesity were significantly lower in participants reporting high-active PA (vs. low- and moderate-active). Physical changes common with aging are weakening of the musculoskeletal system and increased obesity, and loss of bone and muscle mass is associated with fracture risk and decreased body function (Genest et al., 2021). Decreased physical performance with loss of muscle mass is a signature of sarcopenia. The annual mass and performance losses in men over 60 years of age have been reported to be about 1–2% and 3%, respectively, and the combination of sarcopenia and osteoporosis or obesity is believed to exacerbate that effect (Von Haehling et al., 2010; Scott et al., 2017). In this study, the rates of osteoporosis, sarcopenia, obesity, and sarcopenic obesity were higher with low-active PA than with high-active PA, consistent with the results of previous studies (Genest et al., 2021) reporting that increased PA was related to health improvements, such as in osteoporosis and sarcopenia.

The WHO recommends 150 min of moderate-intensity or 75 min of high-intensity PA for more than 3 days a week for the elderly (Bull et al., 2020). PA in older adults helps reduce the incidence of overweight, obesity, and MetS by causing skeletal muscle contraction, and improves musculoskeletal health by increasing muscle strength and flexibility, which are factors related to osteoporosis and sarcopenia (Piercy et al., 2018). A study by Min et al. (2021) reported that higher PA reduced the prevalence of osteoporosis, and even lower PA reduced the prevalence of osteoporosis with higher levels of vitamin D in the blood. Not only PA but also nutrient intake can be an important factor affecting bone formation and maintenance in older people, as they can be attributed to factors such as calcium and vitamin D deficiency, or parathyroid hormone secretion disorders (Onambele-Pearson et al., 2019). Osteoporosis can be effectively prevented and treated by various PAs, such as weightlifting, walking, running, jumping, climbing stairs, swimming, and underwater aerobics, as shown in a review by Moreira et al. (2014). It has also been reported that PA can increase walking speed, balance, and daily life activities in older people with sarcopenia (Steffl et al., 2017), and that it can improve mobility and physical function in older patients (Chou et al., 2012; De Vries et al., 2012). Son et al. (2019) reported that the prevalence of sarcopenia, obesity, and sarcopenic obesity significantly decreased when over 2900 MET-min/week of PA was performed. In this study, moderate-active PA was effective in preventing sarcopenia, and few older adults with obesity and sarcopenic obesity in high-active PA, supporting the results of prior studies. Therefore, it is necessary to develop programs to maintain regular PA among older people with sarcopenia, obesity, and sarcopenic obesity.

This study has the following limitations. First, the PA levels used in this study were based on each participant’s subjective account and may have differed from their actual amount of PA performed. Second, the amount of PA was quantified by cross-sectional surveys of the National Health and Nutrition Survey, not by heart rate measurements and accelerometer measurements; thus, errors may have occurred. Third, although it is possible to confirm the relationship of the amount of PA in Korean elderly people with osteoporosis, sarcopenia, obesity, and sarcopenic obesity, it is difficult to elucidate the causal relationship. Fourth, this study analyzed significant factors related to osteoporosis, sarcopenia, obesity, sarcopenic obesity, and cardiometabolic diseases, such as age, education level, smoking status, and alcohol consumption, but other factors were not considered. Despite the above limitations, the results of this study are thought to be meaningful.

In summary, a statistically significant association was observed between PA levels and cardiometabolic risk factors, including percent body fat, waist circumference and total cholesterol. The associations of PA levels with systolic and diastolic blood pressure, fasting glucose, and low-density lipoprotein cholesterol were not significant. High-active PA (vs. low-active PA) was associated with a lower prevalence of sarcopenia, obesity, sarcopenic obesity, osteoporosis, and cardiometabolic risk factors in both unadjusted and adjusted models. Therefore, we verified a lower prevalence of sarcopenia, osteoporosis, obesity, and cardiac metabolic disease in Korean elderly with more active PA. This suggests that more active PA maybe reduce the prevalence of sarcopenia, osteoporosis, obesity, and cardiometabolic diseases in older adults.

## Data Availability Statement

The raw data supporting the conclusions of this article will be made available by the authors, without undue reservation.

## Ethics Statement

The studies involving human participants were reviewed and approved by the Korea Center for Disease Control and Prevention Institutional Review Board (IRB number: 2008-04EXP-01-C, 2009-01CON-03-2C, 2010-02CON-21-C, and 2011-02CON-06-C). The patients/participants provided their written informed consent to participate in this study.

## Author Contributions

W-SJ, S-WK, and H-YP: conception and study design, statistical analysis, writing—review, and editing. W-SJ and H-YP: investigation and data interpretation. W-SJ: writing—original draft preparation. KL: supervision. All authors have read and approved the final manuscript.

## Conflict of Interest

The authors declare that the research was conducted in the absence of any commercial or financial relationships that could be construed as a potential conflict of interest.

## Abbreviations

ASM: appendicular skeletal muscle mass
CRP: C-reactive protein
DXA: dual X-ray absorptiometry
IGF-1: insulin-like growth factor-1
LDL-C: low-density lipoprotein cholesterol
IPAQ: International Physical Activity Questionnaire
KNHANES: Korea National Health and Nutrition Survey
KDCA: Korea Disease Control and Prevention Agency
MetS: metabolic syndrome
PA: physical activity
(PAI)-1: plasminogen activator inhibitor
SMM: skeletal muscle mass
TEE: total energy expenditure
TG: triglyceride
WHO: World Health Organization.
